# Telomere Length, Oxidative Stress Markers, and Related miRNAs in Non-Invasive Samples of Mild COVID-19 Cases

**DOI:** 10.3390/ijms26104934

**Published:** 2025-05-21

**Authors:** Angélica Domínguez-de-Barros, Candela Sirvent-Blanco, Omar García-Pérez, Malena Gajate-Arenas, Alma García-Ramos, Claudia Migliazzo, José E. Piñero, Jacob Lorenzo-Morales, Elizabeth Córdoba-Lanús

**Affiliations:** 1 Instituto Universitario de Enfermedades Tropicales y Salud Pública de Canarias (IUETSPC), Universidad de La Laguna, 38029 La Laguna, Tenerife, Spain; angelica4arealejos@gmail.com (A.D.-d.-B.); alu0101234656@ull.edu.es (C.S.-B.); ogarciap@ull.edu.es (O.G.-P.); ogajatea@ull.edu.es (M.G.-A.); alu0101204734@ull.edu.es (A.G.-R.); jpinero@ull.edu.es (J.E.P.); 2 Centro de Investigación Biomédica en Red de Enfermedades Infecciosas (CIBERINFEC), Instituto de Salud Carlos III, 28029 Madrid, Spain; 3 Dipartmento di Scienze e Tecnologie Biologiche Chimiche e Farmaceutiche, Universita’ Degli Studi di Palermo, 90133 Palermo, Italy; claudia.migliazzo@gmail.com; 4 Departmento de Obstetricia y Ginecología, Pediatría, Medicina Preventiva y Salud Pública, Toxicología, Medicina Legal y Forense y Parasitología, Facultad de Ciencias de la Salud, Universidad de La Laguna, 38200 La Laguna, Tenerife, Spain; 5 Departmento de Bioquímica, Microbiología, Biología Celular y Genética, Área Biología Celular, Facultad de Ciencias, Universidad de La Laguna, 38206 La Laguna, Tenerife, Spain

**Keywords:** biomarkers, infection response, SARS-CoV2, microRNAs, oxidative stress

## Abstract

Oxidative stress and inflammation influence immune response and epigenetic mechanisms in infectious diseases. In mild COVID-19, host-encoded miRNA profiles remain underexplored, although they reveal mechanistic insights into disease pathogenesis. This study evaluated ageing and oxidative stress biomarkers (telomere length (TL), TBARS, 8-OHdG, and circulating related-miRNA expression) in 75 mild cases and 30 non-COVID-19 controls. TL correlated with age (R = −0.384, *p* = 0.005) and was shorter in cases compared to controls (rTL 1.46 ± 0.51 vs. 0.99 ± 0.37; *p* < 0.001), being similar between saliva and blood samples (*p* = 0.917). miR-138-5p was upregulated in COVID-19 cases (*p* = 0.026) and correlated with 8-OHdG (R = 0.403, *p* = 0.05), which was increased in cases (*p* = 0.040); miR-210-3p was downregulated in infected individuals (*p* = 0.008), while miR-182-5p expression correlated with TBARS (R = 0.582, *p* = 0.018). miR-34a-5p and miR155-5p expression was not altered in mild COVID-19. These findings suggest early systemic cellular damage in mild COVID-19 and highlight miR-138-5p and miR-182-5p as potential early biomarkers of oxidative stress.

## 1. Introduction

COVID-19, declared a pandemic by the World Health Organization (WHO) in March 2020, originated in Wuhan, China, and was caused by the Severe Acute Respiratory Syndrome Coronavirus 2 (SARS-CoV-2). According to the last WHO report, there have been over 775 million confirmed cases and seven million reported deaths worldwide [1]. SARS-CoV-2 is a highly virulent and transmissible virus primarily spread through aerosols. Symptoms can range from asymptomatic to severe outcomes, often accompanied by other aggravating complications such as cardiovascular problems, renal lesions, strokes, and mortality [2]. The virus infects respiratory epithelial cells by binding to the ACE2 receptor via its S protein. This allows the virus to replicate in the cells and migrate to the lungs. It can also be replicated in the ACE2 receptors of other organs (kidney, liver, central nervous system, etc.), for which some consider COVID-19 a systemic disease [3].

In response to the infection, the immune system liberates a cytokine storm cascade, increasing the influx of immune cells and proinflammatory molecules. Cytokine storms are considered the main cause of mortality in COVID-19 cases [4,5].

The phenotypic response to SARS-CoV-2 infection is very heterogeneous. Genetic background plays a significant role in the virus’s pathogenesis and the host’s susceptibility. Studies have reported that SARS-CoV-2 acquires new mutations and virulence factors that could deceive the host’s immune response, finding different profiles on the expression of inflammatory markers in patients with moderate or severe COVID-19 [6]. The different immune profiles associated with the severity of COVID-19 can be assessed by examining molecular, cellular, and immunological approaches in-depth and can significantly contribute to improving disease prognosis [7,8]. In addition to the immune system response, several other factors play a crucial role when studying the progression of COVID-19. These elements are significant in determining the likelihood of developing a severe form of the disease [5,7]. In particular, ageing is a determining factor. The analysis of age-related biomarkers can be useful to determine the possible risks and outcomes of the disease. One principal biomarker of ageing is telomere length (TL) [9]. Telomeres are conserved DNA-tandem-repeated sequences (5′-TTAGGG-3′) located at the end of chromosomes, responsible for maintaining genome stability. Telomere shortening occurs naturally with each cell division [10]. However, infectious processes can intervene and accelerate this shortening. When telomeres reach a critical length, cells enter a senescent state, significantly contributing to ageing and the development of age-related diseases [11]. A dysregulated immune response or an accumulation of pro-inflammatory cytokines can increase genome instability, leading to further telomere shortening and DNA damage that, in turn, may trigger uncontrolled activation of cell death pathways [12].

Telomere shortening has been reported as an indicative marker of disease progression. Shorter TL has been found in individuals with other pulmonary diseases and age-related conditions, like cardiovascular or neurodegenerative affections [13,14]. Concerning COVID-19, several studies have reported that severe and post-COVID-19 survivors had shorter telomeres than non-COVID patients [15]. However, there is no clear evidence of the individual’s response to SARS-CoV-2 infection in patients with only mild symptoms or regarding other related senescence outcomes [16,17].

Another proposed biomarker of cellular senescence and oxidative damage is 8′-hydroxy-2′-deoxyguanosine (8-OHdG), one of the main products of DNA damage resulting from DNA damage caused by reactive oxygen species (ROS), leading to DNA hypomethylation and genomic instability [18]. The biological alterations in 8-OHdG levels have been studied in a wide range of diseases, and its increase has been reported as a prognostic marker of carcinogenesis or age-related features in different neurodegenerative or cardiovascular conditions [19]. Nevertheless, the specific role of 8-OHdG and its impact on the inflammatory response and molecular damage in mild COVID-19 disease needs further investigation, as only a few studies have found significant differences in this marker in severe patients compared to moderate ones [20].

Also, an increase in oxidative stress has been associated with several chronic disorders and COVID-19 [21]. ROS targets, accumulates, and damages other crucial biomolecules, like proteins and lipids. Malondialdehyde acid (MDA) is one of the aldehyde molecule products generated by the breakage of polyunsaturated fatty acid chains in the cell membrane [22]. Other studies have also investigated other oxidative biomarkers in different COVID-19 patients with severe outcomes but could not establish mortality-predictive findings [23,24]. Oxidative stress is acknowledged as an important factor that impacts the severity of COVID-19 [25]. It contributes to the inflammation and cellular senescence associated with the disease. Oxidative stress levels fluctuate throughout the disease, suggesting that different oxidative stress biomarkers should be regularly monitored as early prognostic indicators.

MicroRNAs (miRNAs) are short, noncoding RNAs that have been proposed as interesting biomarkers of disease diagnosis and prognosis. They regulate gene expression, inhibiting or stimulating the translation of mRNA targets. miRNAs are involved in a diverse range of biological pathways and processes, including those related to infectious diseases like COVID-19. In respiratory infections, miRNAs are essential in the host’s response to fight against the infection; however, viruses can alter the levels of miRNAs, making the cells more favourable for viral replication [26]. Some miRNAs have been described in other respiratory illnesses to have a role as early biomarkers of disease [27,28]. Identifying an altered profile of circulating miRNAs in mild COVID-19 might improve our knowledge of SARS-CoV-2 pathogenesis and the diversity of its clinical manifestations.

Some miRNAs of interest, like *hsa-miR-155-5p*, *hsa-miR-138-5p*, and *hsa-miR-34a-5p*, have been associated with proinflammatory, oncogenic, and cell proliferation processes [29,30,31]. The miR-155-dependent reduction of TRF1 expression has been associated with telomere dysfunction, increased telomere damage, and chromosome instability in human breast cancer [32]. In COVID-19, miR-155-5p has been reported as a candidate for regulating immune responses against viral infections, including adaptive and innate immunity [33]. In addition, increased miR-155 expression levels were associated with an increased risk of COVID-19 severity and mortality [34], showing the lack of studies on patients with mild forms of the disease to complete the scenario. miR-34a-5p has been reported to have a diagnostic value for patients with Parkinson’s disease [35]. McDonald et al. (2021) found that the levels of miR-155-5p and miR-34a-5p within seven circulating miRNAs were decreased in patients with COVID-19 [36]. A few studies have reported miR-138-5p as a possible target for therapeutic miRNA approaches in COVID-19 [37].

On the other hand, *hsa-miR-210-3p* and *hsa-miR-182-5p* have been associated with oxidative stress and immune responses. miR-210-3p has been reported to be majorly implicated in hypoxic responses and associated with various targets involved in neuroinflammation, lipid metabolism, and diseases such as Parkinson’s disease, hepatocellular carcinoma, atherosclerosis, etc. [38]. In COVID-19-related studies, miR-210-3p has been reported to be lung-specific, targeting some SARS-CoV-2 genes in hypoxic conditions [39]. miR-182-5p has been identified as a marker with prognostic potential in cancer biology. Meanwhile, to our knowledge, the expression and molecular implications of miR-182-5p in mild COVID-19 patients have not been assessed.

All the hallmarks named previously are interconnected and could play a fundamental role in interacting with the viral processes of SARS-CoV-2. Understanding interactions between the occurring inflammatory response in COVID-19 and host ageing characteristics may be useful in assessing the different risks of developing serious forms or consequences of the infection in individuals.

The primary objectives of this study are to evaluate a set of biomarkers, such as telomere shortening and oxidative stress indicators (TBARS and 8-OHdG), in non-invasive samples among COVID-19 patients with mild symptoms and study telomere and oxidative stress-related miRNA expression in these cases to give insights into their potential as promising diagnostic and prognostic targets and help characterise the main aspects of mild infection.

## 2. Results

**Figure 1:**
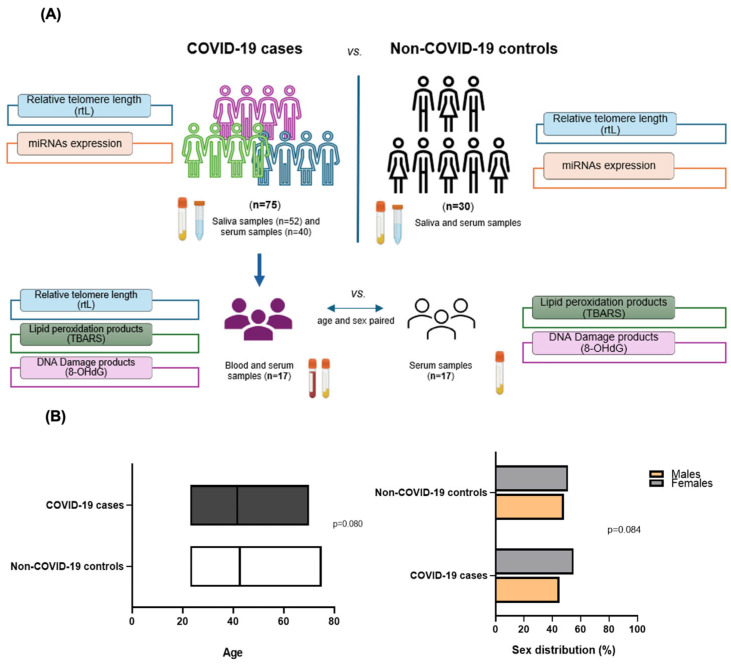
Selection of the individuals included in the study and sampling methods. (**A**) Diagram illustrating the selection and assessment of COVID-19 patients and non-COVID-19 controls included in the study and the biomarkers measured in each group. (**B**) Age (**left**) and sex (**right**) distribution of the whole cohort of the study.

The study’s cohort characterisation is represented in Figure 1A. In the first step of the study, 105 individuals were analysed, and then a subcohort of 34 individuals (17 COVID-19 cases age- and sex-matched with 17 non-COVID-19 individuals) was used for validation. In cases where they presented mild symptoms (fever, headache, cough, etc.). The average age of the whole study cohort was similar between cases and controls (42.56 years for the control group and 41.62 years for COVID-19 cases, *p* = 0.080). No differences were detected in terms of sex in the studied groups (*p* = 0.084) (48.1% men and 51.9% women vs. 45% men and 55% women in controls and cases, respectively) (Figure 1B). The baseline and clinical characteristics of the participants included in the study are represented in Table S2.

### 2.1. Telomere Shortening

**Figure 2:**
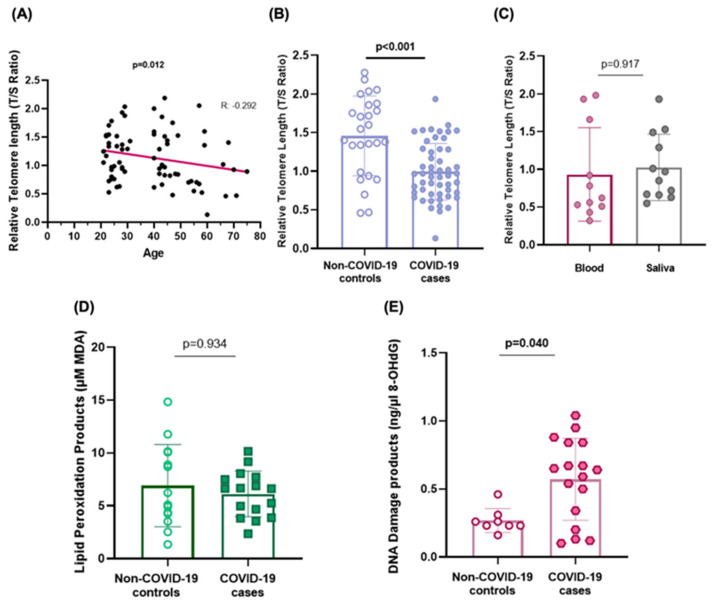
Ageing and oxidative stress biomarkers in mild COVID-19. (**A**) Correlation between saliva relative telomere length (T/S) and age in the whole cohort of the study (Pearson’s correlation test). (**B**) Relative telomere length (rTL) determined in saliva samples in controls vs. COVID-19 cases (T-test for independent samples). (**C**) Relative telomere length (rTL) measured in different tissues of COVID-19 cases (*T*-test for paired samples). (**D**) Serum MDA levels and (**E**) 8-OHdG levels evaluated in COVID-19 cases vs. age- and sex-paired non-COVID controls. The differences in the median (25–75 pc) of TBARS and DNA damage between COVID-19 cases and non-COVID individuals were assessed by the U-Mann–Whitney test. *p*-values < 0.05 were considered significant.

From the COVID-19 cases, saliva samples available from 52 patients were used to assess telomere length. Relative telomere length (rTL) determined in saliva samples was observed to shorten progressively with age (R = −0.292, *p* = 0.012) (Figure 2A). This relationship was found for individuals with a COVID-19 diagnosis (R = −0.384, *p* = 0.005) as well as for healthy controls (R = −0.384, *p* = 0.071) (Figure S1). Shorter telomeres (rTL) were observed in the saliva of individuals with mild COVID-19 when compared to controls without the infection (0.99 ± 0.37 vs. 1.46 ± 0.51, *p* < 0.001, respectively) (Figure 2B). Telomere length shortening was associated with an increased risk of COVID-19 (OR = 0.051, 95% CI = 0.011–0.236; *p* < 0.001, adjusted by age and sex in a logistic regression analysis). Additionally, rTL values observed in saliva were similar to those observed in blood tissue of the same individuals in the group of COVID-19 cases (0.930 ± 0.6 vs. 1.026 ± 0.4, respectively, *p* = 0.917) telomere length was similar among the infected individuals that showed low and medium.(Figure 2C).

Telomere length was similar among the infected individuals that showed low, medium, or high viral loads (Ct) (*p* = 0.859).

### 2.2. Biomarkers of Oxidative Stress

The oxidative stress biomarkers were measured in serum samples of the subcohort of SARS-CoV-2-infected individuals with paired controls (Figure 1A).

When analysing the different biomarkers, neither TBARS nor 8-OHdG seemed to correlate with age (R = 0.175, *p* = 0.354 and R = 0.203, *p* = 0.341, respectively). Also, no differences were observed in the TBARS or 8-OHdG levels between sexes [median (± 25–75 pc); 6.76 (4.89–10) in men vs. 4.91 (2.99–8.28) in women, *p* = 0.089 for TBARS, and 0.46 (0.46–0.67) in men vs. 0.37 (0.37–0.79) in women, *p* = 0.842 for 8-OHdG, respectively]. The serum concentration of lipid peroxidation products (TBARS) was similar in mild COVID-19 cases when compared to non-COVID-19 controls [median (± 25–75 pc); 6.63 (4.07–7.66) vs. 6.23 (3.89–10.11), *p* = 0.934, respectively] (Figure 2D). On the contrary, COVID-19 cases accumulated higher serum concentrations of DNA damage products than individuals who did not suffer the viral infection [median (± 25–75 pc); 0.64 (0.27–0.849) vs. 0.23 (0.23–0.26) ng/mL of 8-OHdG, *p* = 0.040] (Figure 2E). The logistic regression analysis suggests an increased risk of disease in those individuals with augmented DNA damage (OR = 3.56, 95%CI = 1.02–12.24; *p* = 0.049).

### 2.3. Telomere and Oxidative Stress-Related microRNA Expression

Among the subset of analysed miRNAs, miR-34a-5p expression levels were found to correlate with age in controls (R = 0.594, *p* = 0.042). Both miR-138-5p and miR-34a-5p were highly expressed in women when compared to men in the control group (*p* = 0.041 and *p* = 0.009, respectively).

**Figure 3:**
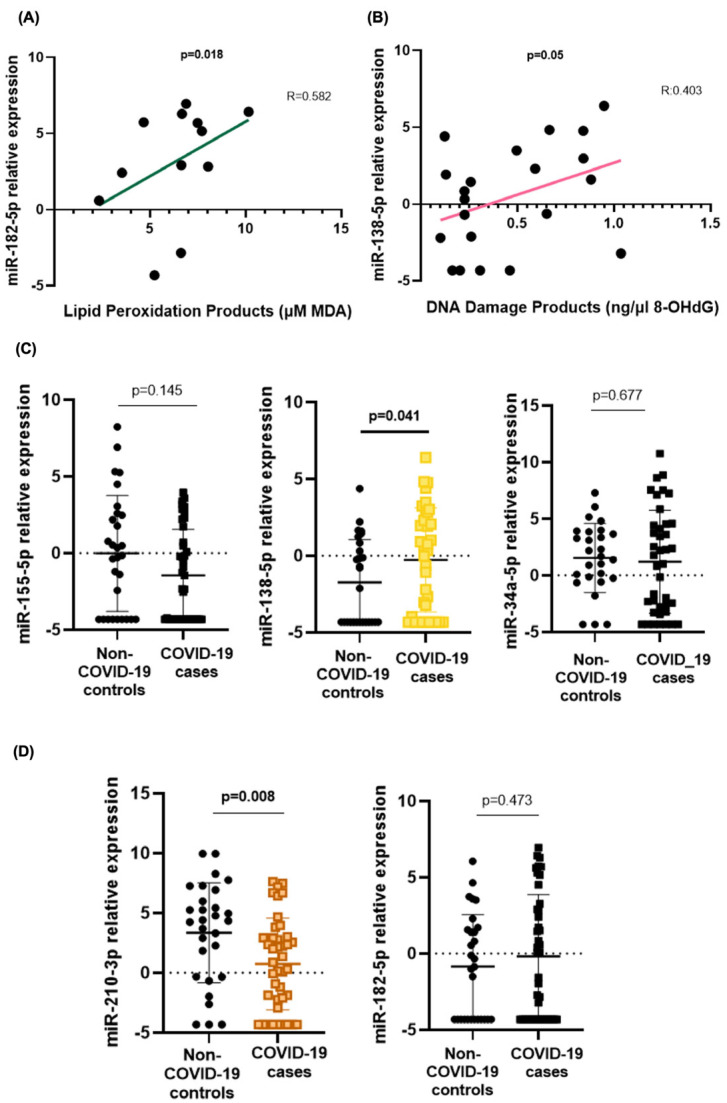
Telomere- and oxidative stress-related microRNA expression in mild COVID-19. (**A**) Correlation between miR-182-5p and MDA levels. (**B**) miR-138-5p expression and 8-OHdG concentrations in COVID-19 cases. (**C**) Differential telomere-related miRNA expression between COVID-19 cases and controls (from left to right, miR-155-5p, miR-138-5p, and miR-34a-5p). (**D**) Differential oxidative stress-related miRNA expression between COVID-19 cases and controls (miR-210-3p and miR-182-5p). Spearman’s coefficient correlation was performed to evaluate the relationship between the stress biomarkers and the selected miRNAs. Differences in the expression of miRNAs between cases and controls were determined by the U-Mann–Whitney test. *p*-values < 0.05 were considered significant.

miR-182-5p expression positively correlated with age in COVID-19 patients (R = 0.520, *p* = 0.047). An increase in lipid peroxidation products was found to be related to an increase in the expression of miR-182-5p (R = 0.582, *p* = 0.018) (Figure 3A).

Elevated 8-OHdG concentrations were found to be correlated with an increased expression of miR-138-5p in overall cases and controls (R = 0.403, *p* = 0.05) (Figure 3B). Among all the circulating miRNAs analysed, miR-138-5p was the only one significantly overexpressed in mild COVID-19 cases compared to non-COVID-19 controls (*p* = 0.041) (Figure 3C).

On the other hand, miR-210-3p was significantly downregulated in individuals with the viral infection (*p* = 0.008) (Figure 3D).

### 2.4. Functional Annotation Analysis and Gene Target Prediction

The in-silico functional analysis using KEGG pathways revealed that the analysed microRNAs are involved in various signalling pathways (Table 1). miR-34a-5p, miR-138-5p, and miR-182-5p were found to participate in viral carcinogenesis, as well as other cancer-related processes. miR-34a-5p also participates in cell cycle pathways, being especially related to MAPK signalling. miR-210-3p also participates in cancer processes by interacting with other microRNAs. On the other hand, miR-155-5p is related to the immune system and inflammatory process.

Furthermore, enrichment analysis performed using GO terms focused on the biological processes associated with the included miRNAs is presented in Table S3. Most notably, all miRNAs except miR-210-3p showed their participation in viral processes (GO: 0016032), stress response (GO: 0006950), gene expression (GO: 0010467), and biological processes involved in symbiotic interaction (GO: 0044403). In addition, miR-34a-5p is particularly implicated in the stress-activated MAPK cascade (GO: 0051403, *p* = 6.65 × 10^−8^) and the viral life cycle (GO: 0019058, *p* = 1.39 × 10^−4^). Meanwhile, miR-138-5p plays a role in processes such as the mitotic cell cycle (GO: 0000278, *p* = 3.71 × 10^−7^) and immune system process (GO: 0002376, *p* = 1.32 × 10^−3^), the same as miR-155-5p (*p* = 1.26 × 10^−5^). miR-182-5p is linked to the mRNA metabolic process (GO: 0016071, *p* = 4.51 × 10^−17^) and cell death (GO: 0008219, *p* = 1.11 × 10^−17^). On the other hand, miR-210-3p was related to other GO terms like organelles instead (GO: 0043226, *p* = 5.22 × 10^−3^) and ion binding (GO: 0043167, *p* = 4.29 × 10^2^).

## 3. Discussion

The different outcomes of COVID-19 require different approaches, from both medical and research perspectives, to enhance understanding of the disease’s progression and to identify potential methods for preventing the characteristic excessive inflammatory response. This study explored the diverse host response to SARS-CoV-2 infection by examining molecular biomarkers regarding inflammation, oxidative stress, DNA damage, and ageing in noninvasive samples of patients with mild COVID-19. This study aimed to fill the knowledge gap, particularly in mild cases, to better understand the implications for patient outcomes.

The main findings of our study highlight the significance of shorter telomere lengths and increased DNA damage levels in mild COVID cases compared to controls as early indicators of the disease. This study identified key expression patterns of miRNAs like miR-210-3p and miR-138-5p, suggesting their role in infection response and cellular damage regulation in mild cases.

Infectious diseases are also associated with premature ageing due to senescence events that occur in the cells in response to various stress stimuli, such as DNA damage, telomere shortening, oncogene activation, paracrine secretion, etc., that further promote these pathological processes [40]. Variability in telomere length is considered an important parameter for understanding disease progression [41]. Moreover, as already known, oxidative stress could enhance telomere attrition [42]. In the case of viral infections, shorter telomeres are typically observed in infected patients compared to healthy individuals. Shorter telomeres have been previously observed in patients with severe COVID-19 [15]. However, little information exists regarding telomere dynamics in patients with a mild course of the disease, nor have significant results been found [43]. In our study, mild COVID-19 cases exhibited shorter telomeres when compared to age-matched controls without the viral infection. This finding is indicative of the early damage produced to the main biomolecules in the organism, which may lead to immunosenescence, chronic inflammation, or a detrimental response to fight the infection. Additionally, most published research focuses on measuring rTL from blood leukocyte samples, which are typically less accessible and may carry a higher risk of clotting or hospital-acquired infections. Measuring this marker in non-invasive tissue like saliva has been scarcely performed until recently. In this research, we assessed rTL in blood and saliva samples, finding similar telomere length values in both tissues. This fact indicates that saliva is a valid sample for this type of study and is representative of the reference tissues, as stated in previous research [44].

Increased oxidative stress production is common in viral and other infectious diseases, like bacterial pneumonia and COVID-19 [45]. The permanent production of ROS damages many biomolecules and could modulate the immune response and the epigenetic machinery through the relationship between several inflammatory components and microRNAs. This affects the immune system’s ability to effectively combat and resolve the infection from early to later stages while avoiding detrimental inflammation. Among the different reactive oxygen species (ROS) produced, persistently elevated plasma TBARS levels have been reported in some clinical processes involving some grade of inflammation [46]. In previous studies, a significant increase in TBARS has been reported among patients infected with SARS-CoV-2, ranging from those with moderate symptoms to severe cases [46]. To our knowledge, only one study reported elevated PMBC levels of lipid peroxidation products in mild COVID-19 patients compared to healthy controls [47]. However, we did not observe these significant changes, at least in the mild COVID-19 cases studied. Nevertheless, our findings are consistent with those reported by Cekerevac et al. (2021), where patients exhibited similar levels of TBARS independent of disease severity [48].

Focusing on DNA damage, we have found higher 8-OHdG serum concentrations in COVID-19 patients than in non-COVID-19 controls, although inconsistent results have been found in several studies [24]. The association of shorter telomeres and increased DNA damage found in our study in mild COVID-19 disease could indicate systemic cell damage processes in patients even at the early stages of the disease. Therefore, these markers are valuable for assessing the severity of SARS-CoV-2 infection and its evolution.

Taking into account the changes that occur at the genetic and epigenetic expression level, we found that miR-182-5p expression correlated with increased lipid peroxidation in COVID-19 individuals. In addition, our in silico analysis supports this finding by revealing miR-182-5p to be involved in fatty acid biosynthesis and metabolic processes. In this context, some specific target genes of miR-182-5p, like *GPX4*, *SLC7A11*, *FTH1*, and *SAT1*, as pointed out by the predictive analysis, may be of interest. Jankauskas et al. (2023) reported that patients who did not survive the infection suffered an increase in lipid peroxidation levels measured by markers (like 4-hydroxynonenal (4-HNE) and C11-BODIPY) [49]. However, these results have been observed in studies involving severe patients, and considering the dynamic of lipid peroxidation in the disease, TBARS products were particularly significant during this stage [46]. In our study, we did not find significantly increased MDA products in mild cases with respect to non-COVID-19 controls, suggesting that changes in lipids measured in plasma at the product level (MDA) may not be significantly noticeable at the early stages of the disease. However, changes in the regulation of this pathway could be detectable, as suggested by our findings. This relationship at the early stages of the infection could be linked to the disease outcome.

On the other hand, miR-138-5p is generally involved in the negative regulation of gene expression and inflammatory responses. In particular, bioinformatics predictions indicate that miR-138-5p has an antiviral role in HIV and HSV-1 infection, so it might also target SARS-CoV-2 genes [50]. This could explain our finding of overexpression of miR-138-5p in mild COVID-19 patients since it may be acting directly against certain targets of the virus to help resolve the infection, being observable even at very early stages of the disease. This finding could help understand the potential role of certain miRNAs in regulating host-virus interactions during infections. Host cells can use miRNAs to limit viral replication by targeting viral genomes or transcripts directly or indirectly by modulating host factors involved in the immune response [50]. Furthermore, miR-138-5p also correlated with elevated DNA damage products. As the most common viral infection requires the breakage of the host DNA, we could find genomic instability and DNA damage in infected patients [51]. The increased expression of miR-138-5p could be a mechanism to act against any possible damage produced during the infection.

Scarce information has been published regarding miR-210-3p and COVID-19. In our study, decreased levels of expression were found in individuals with symptoms. miR-210-3p has been described as a lung-specific miRNA, playing a potential role in SARS-CoV-2 pathogenesis by modulating the response to hypoxia and inflammatory conditions [39]. In previous work from our group, we have already reported that miR-210-3p was found to be downregulated in COVID-19 patients undergoing a mild infection, which reinforces its role, as these patients did not have to face hypoxic conditions [52]. A recent study reported miR-210-3p as a regulator of hub genes potentially involved in the association between venous thromboembolism and COVID-19 [53]. Moreover, our in silico analysis pointed out miR-201-3p to target *E2F3* (E2F transcription factor 3), which is known to act as a crucial protein in the cell cycle process. Interestingly, it was described that the overexpression of miR-210-3p was reported in lung, ovarian, and pancreatic cancer cells, which directly targets *E2F3*, linking hypoxia with the regulation of the cell cycle [54].

Importantly, the study by Wu et al. (2021) reported that certain transcription factors, including STAT5B, STAT3, STAT6, and E2F3, among others, were strongly connected with IFN or IFN receptors in asymptomatic individuals with SARS-CoV-2 infection [55]. These authors confirmed that in critical patients, the IFN response failed by measuring the expression of IFN-stimulated genes. It is already known that an effective IFN response can eliminate viral infection [56].

The senescence process also involves the activation/regulation of different miRNAs, some of them known as “inflamma-miRs”, like miR-34a-5p, miR-150-3p, miR-155, etc. [57]. Our results showed that miR-34a-5p is increased with age. Our in silico analysis confirmed that this miRNA is set to play a role in both the cellular cycle and fatty acid pathways. Therefore, the intervention of this miRNA may contribute to cellular mechanisms naturally involved in the telomere attrition that occurs in the ageing process. An overexpression of this miRNA was found to inhibit telomerase activity and induce signalling for cell arrest or apoptosis in cancerous processes [58]. miR-34 levels are increased in neurodegenerative disorders and age-related cardiovascular diseases [59,60]. miR-34a targets *SIRT1* and reduces its expression, which is especially important as Sirtuin 1 protects against age-related diseases, especially metabolic ones [61]. Regarding COVID-19, Saulle et al. (2023) reported upregulated levels of miR-34a-5p in both plasma and saliva of severe COVID-19 patients [62]. In mild patients, they also detected elevated levels of this miRNA in saliva samples, suggesting that the modulation of this miRNA occurs even at low viral replication rates but is locally focused on the infected tissue [62]. We found similar levels of expression of miR-34a-5p in serum samples of patients with mild COVID-19 with respect to controls, which supports the hypothesis that miR-34a-5p overexpression is a marker of COVID-19 severity and poorer outcomes.

Studies among COVID-19 patients showed that miR-155-5p was generally upregulated in patients experiencing a more severe disease compared to mild cases or healthy individuals [34,63]. As reported in the *in-silico* analysis, miR-155 plays a critical role in viral diseases by modulating antiviral responses, including inflammatory and immune responses. It appears that the expression of this miRNA peaks during the acute phase of the infection, making it one of the first miRNAs to be stimulated in the immune response, which can trigger a cytokine storm. Then, it gradually decreases during the negative-turn phase of infection and recovery [64]. Therefore, it may be the case in our study, where no dysregulation in the expression of miR-155-5p was observed in mild cases when compared to controls, due to the absence of exacerbated inflammation. Other studies have reported that moderate overexpression of miR-155 could contribute to the severe inflammatory state of this disease or other malignancies [63]. According to this, the dynamics of miR-155-5p expression could be a good candidate marker for studying the progression of COVID-19 severity. Our findings contribute to understanding the behaviour of this miRNA before a harmful inflammation is reached, as an “inflection point”. Donyavi et al. (2021) studied the effect that an anti-miR-155 may cause in the lungs of infected patients, reporting attenuated inflammation and suggesting new therapeutic strategies against COVID-19 [64].

As previously detailed, the role that oxidative stress and inflammation play in modulating the immune response and epigenetic machinery in the pathogenesis of SARS-CoV-2 is noteworthy. Also, the relationship between oxidative stress and inflammation with the different subsets of miRNA profiles has gained increasing interest recently [57]. Taken together, these findings indicate the importance of evaluating these molecules (TL, oxidative stress products, and miRNAs) as hallmarks to characterise and predict inflammatory response, ROS production, and cell senescence, among others, regarding COVID-19 disease progression.

### Limitations of the Study

Even so, this study has some limitations. The determination of the different stress biomarkers was performed in serum, so levels of expression may differ between different types of tissues during the immune response. In the same way, saliva was validated as a useful tool for measuring biomarkers such as telomere length. In this regard, recent studies have reported the diagnostic utility of saliva in detecting cardiovascular diseases (CVD), systemic and local inflammation, and endocrinological and metabolic disorders [65]. Nevertheless, further studies measuring the significant biomarkers identified in this research in specific tissues would be beneficial to confirm the correlation of their expression across different tissues. Using non-invasive, clinically accessible samples such as saliva and blood, rather than representing a limitation, represents a significant strength, particularly in the context of longitudinal patient monitoring and early disease stratification. Secondly, as our study focused on mild COVID-19 cases, it may be necessary to perform a broader study of the expression of the included biomarkers in a larger cohort, also including severe patients, to confirm our findings. Monitoring severe cases from the onset of the first symptoms should provide valuable insights into the mechanisms involved in viral pathogenesis and its progression to severe or critical forms. Third, the sample collection procedure covered a period where all diagnosed individuals were infected with the Omicron variant. Since it is important to report the variants of SARS-CoV-2 that could cause differences in the dysregulation of immune genes or biomarkers of stress. Lastly, we acknowledge that further functional examination in vitro, including transformed cell lines and primary human lung cells, is necessary to confirm the specific targets of the proposed miRNAs identified in this research. Moreover, we would like to emphasise that our research is based on the analysis of clinical samples from individuals with mild COVID-19, an underexplored subgroup, using gold-standard techniques further supported by an in-depth bioinformatics analysis to explore potential targets and pathways. This bioinformatics-driven prioritisation analysis is commonly used as a foundation for future mechanistic studies.

Nevertheless, our findings are worthy as a preliminary approach to better understand the disease in patients with mild conditions and can be used to predict further possible severity progression. In this context, the possibility of exploiting non-invasive biomarkers as a tool to detect systemic disorders may constitute an intriguing opportunity to implement strategies to diagnose and follow up patients affected by different disorders [65]. Another interesting pathway to investigate is the possibility of decreasing ROS production as a tool to mitigate SARS-CoV-2 complications. This signifies the importance of early immunological interventions that target inflammatory markers, which are predictive of severe symptoms, rather than targeting the late-appearing cytokine storm outcomes. In this scenario, to our belief, the inclusion of patients with a mild course of the disease in population studies, as well as performing longitudinal studies with a follow-up of patients who may develop severe forms of the disease, plays a key role in the investigation for early intervention.

In conclusion, our findings of a shorter telomere length and an increase in oxidative stress products in mild COVID-19 cases are indicative of the early systemic cellular damage processes present in mild infections caused by SARS-CoV-2. Regarding the analysed profile of telomere length and oxidative stress-related miRNAs, the overexpression of miR-138-5p in mild COVID-19 cases may be indicative of an active compensatory mechanism against the infection. In addition, miR-182-5p might serve as an early biomarker of oxidative stress in COVID-19. The role of miR-155-5p and 34a-5p in disease severity was confirmed. Future prospective studies in a larger cohort are needed to confirm present findings.

## 4. Materials and Methods

### 4.1. Selection of Individuals

The study comprised a total of 105 individuals, of whom 75 were cases with a molecular diagnosis in the upper airways of infection by SARS-CoV-2 presenting mild symptoms of the disease, and 30 individuals without present or past infection named as non-COVID-19 and corresponded to controls. The included individuals were recruited from a population screening study at the University of La Laguna and private laboratories in Tenerife, Spain, between 2020 and 2022. A subsample of 17 individuals from the whole mild COVID-19 cohort were age- and sex-paired with another group of 17 non-COVID-19 individuals included as controls for the analysis of blood biomarkers (TL and oxidative stress products) (Figure 1A). All the individuals included in the study were fully vaccinated at the time of sample collection.

As inclusion criteria, cases with mild forms of COVID-19 that did not require more than ambulatory care were included. The severity of COVID-19 was classified according to NIH guidelines [66] (Mild illness: People who have any of the various signs and symptoms of COVID-19 (e.g., fever, cough, sore throat, malaise, headache, muscle pain, nausea, vomiting, diarrhoea, loss of taste and smell) but do not have difficulty breathing, dyspnoea, or abnormal chest imaging. Subjects presenting with any other respiratory diseases or comorbidities or those receiving medical treatment were excluded from this study.

The Hospital Universitario de Canarias ethical committee board approved the study (CHUC B1947), and written informed consent was obtained from all participants. The study was conducted in accordance with the Declaration of Helsinki.

### 4.2. Sampling

Biological samples were collected during the first seven days of infection or at the beginning of symptoms for virus detection. These samples consisted of spitted saliva and/or peripheral blood that were used to analyse the proposed biomarkers (Figure 1A). Saliva was collected after stimulating the buccal area for around two minutes in a sterile container and conserved at 4 °C for immediate nucleic acid isolation.

When performing blood withdrawal, it was collected in tubes with EDTA. Whole blood and serum were separated after centrifugation at 3000 rpm for 15–20 min and stored at −20 °C. Blood cells were further processed for DNA isolation.

### 4.3. Telomere Length Measurement

DNA from saliva samples was extracted using the Maxwell^®^ 16 Viral Total Nucleic Acid Purification Kit (Promega TM Corporation, Madison, WI, USA) following the manufacturer’s instructions and processed in the Maxwell^®^ 16 instrument (Promega, Madison, WI, USA). DNA extraction from whole blood was performed using the TRIzol^®^ Reagent (Ambion Life, Austin, TX, USA) following the manufacturer’s instructions.

Relative telomere length (rTL) was determined in saliva and/or blood samples by qPCR following the conditions established by Cawthon (2009) [67]. This method consists of comparing the copy number of the telomere gene (T) to a reference single-copy gene (S) in each sample. rTL was obtained using the (T/S) ratio by the ΔΔCt method and corrected using the method established by Pfaffl (2001) [68]. Albumin (*ALB*)was used as a reference gene. qPCR reactions and conditions have been reported in previous research by our group [13] (Table S1). Internal controls for long and short telomeres were used. Samples were evaluated in duplicate and analysed in a Step-One Plus real-time PCR thermocycler (ThermoFisher Scientific Inc.,Waltham MA, USA). The mean plate efficiency was 98% for the telomere and 90% for the albumin assays.

### 4.4. Oxidative Stress Biomarkers Measurement

Products of oxidative metabolism, like malondialdehyde (MDA) and thiobarbituric acid (TBARS), were determined in 20 μL of serum samples using the OxiSelect TBARS Assay Kit (Cell Biolabs, Inc., San Diego, CA, USA) following the manufacturer’s instructions. Each sample was evaluated in duplicate. The formed adduct of MDA-TBA was measured fluorometrically at 540nm excitation and 590 nm emission on the EnSpire Multimode Plate Reader (Perkin-Elmer, Madrid, Spain). Quantification of MDA (µM) was obtained through a Standard Curve of known concentrations.

The determination of 8-hydroxy-2′-deoxyguanosine (8-OHdG) was assessed using the OxiSelectTM Oxidative DNA Damage ELISA Kit (8-OHdG Quantitation) (CellBiolabs, San Diego, CA, USA) following the manufacturer’s instructions. A total of 50 µL of serum samples diluted 1:20 were used in duplicate in each assay. The absorbance was determined spectrophotometrically at 450nm on the EnSpire Multimode Plate Reader (Perkin Elmer, Madrid, Spain). The 8-OHdG quantification (ng/µL) was determined by the comparison of a 4-parameter-logistic line Standard Curve, using the SigmaPlot 12.0 program (Systat Software, 2010 Inc., San Jose, CA, USA).

### 4.5. miRNAs Validation by qPCR

miRNA isolation from serum samples was performed using the miRNeasy Serum/Plasma Advanced Kit (Qiagen, Hilden, Germany). Then, miRNAs were retro-transcribed into cDNA using the miRCURY LNA RT Kit (Qiagen, Hilden, Germany).

The expression of miR-155-5p, miR-138-5p, miR-34a-5p, miR-210-3p, and miR-182-5p was evaluated by qPCR using the miRCURY LNA SYBR Green PCR Kit (Qiagen, Hilden Germany). As a reference gene, miR-26a-5p, which was previously determined as the best choice based on its stability in these particular samples, was used in each assay [52]. Each reaction was performed in duplicate and was set up for gene expression quantification in a StepOne Plus thermocycler (ThermoFisher Scientific, Waltham, MA, USA). The relative expression analysis of the target genes was determined using the comparative threshold method 2^ΔΔCt^.

### 4.6. miRNAs In Silico Analysis

The predicted targets, cellular functions, and metabolic pathways of the miRNAs included in this study were assessed through computational prediction across different software: TargetScan Human v.8 (https://www.targetscan.org/vert_80/) (accessed on 29 November 2024), miRDB (https://mirdb.org/mirdb/index.html) (accessed on 29 November 2024), and miRbase (https://www.mirbase.org/) accessed on 29 November 2024). Additionally, DianaTools miRPath v.3 (https://dianalab.e-ce.uth.gr/html/mirpathv3/index.php?r=mirpath) (accessed 20 February 2025) was used to investigate the molecular functions and pathways associated with these miRNAs, specially focused on viral infections. Functional and enrichment analyses were performed through Gene Ontology (GO) and the Kyoto Encyclopaedia of Genes and Genomes (KEGG), respectively.

### 4.7. Statistical Analysis

Data are represented by their mean ± standard deviation (SD). The normal distribution of the variables was assessed by the Kolmogorov–Smirnov and Shapiro–Wilkinson tests when required. Variables such as *lipid peroxidation products* (TBARS) and *DNA damage* (8-OHdG) were log10 transformed for normalisation. These variables are represented by their median ± (25–75) percentiles. miRNA relative expression is represented by the Log2 of the expression ratio (2^ΔΔCt^) for normalisation.

The variable *viral load* was divided into tertiles according to the threshold cycle (Ct) of the molecular diagnosis of SARS-CoV-2 by qPCR, considering a low load (Ct > 30), medium load (24 > Ct > 30), and high load (Ct < 24).

Cases and controls were paired by age and sex. The influence of the sex and viral load factors on the study variables was tested by one-way ANOVA.

Pearson’s and Spearman’s correlations were used to evaluate the relationship between the variables depending on normality. Pearson’s correlation test was used to assess the correlation between *telomere length* and *age,* while the oxidative stress variables (*TBARS*, *8-OHdG*) and *miRNA expression* were assessed using Spearman’s test. Subsequently and under these criteria, variable comparisons between the different groups were performed using T-tests and U-Mann–Whitney or Kruskal–Wallis tests.

Logistic regression analysis was performed, controlling for age and sex. A T-paired sample test was performed to compare relative telomere length (rTL) in different tissues (saliva and blood) from the same individuals in the COVID-19 individuals’ subcohort.

Statistical analyses were performed in SPSS 25.0 IBM software and considered significant for *p*-values < 0.05. Graphs were designed with GraphPad Prism v9.0 (Dotmatics, GraphPad Software, San Diego, CA, USA).

## Figures and Tables

**Table 1:** Pathway enrichment analysis of the studied miRNAs (KEGG pathways).

KEGG Pathways	Genes	*p*-Value
	**miR-34a-5p**	
Fatty acid biosynthesis	*FASN, ACSL4, ACSL1, ACACA*	9.94 × 10^−8^
Cell Cycle	*CDC6, CDKN2C, E2F1, CCNB1, SFN, CDK4, E2F2, CCNA2, HDAC1, MCM6, YWHAG, ORC1, MCM4, FZR1, MCM5, STAG2, CDKN2B, STATG1, CDK1, CDKN2A, CDK6, TGFB1, MCM7, TP53, ATM, CCND1, SMAD4, E2F5, E2F3, CDC23*	8.76 × 10^−5^
Pathways in cancer	*BRAF, STAT3, PDGFRA, E2F1, ERBB2, NFKB1, MET, ADCY1, FIGF, GNA12, ROCK1, CK4, CXCL8, RAC2, E2F2, ADCY7, FGFR3, CRKL, STK4, MAP2K2, BIDM, AGTR1, GNAS, LAMA5, CRK, RUNX1, PIK3CB*	2.00 × 10^−4^
Fatty acid metabolism	*ASN, SCD5, ACOX1, ACOX3, ACAA2, PTPLA, CPT2, ACADVL, SCD, ACSL4, ACSL1, HSD17B12, MECR, ACACA*	2.13 × 10^−4^
Viral carcinogenesis	*STAT3, NFKB1, GTF2E2, CDK4, RASA2, CCNA2, GTFH1, PIK3CB, HDAC7, HDAC1, DLG1, VAC14, LTBR, PIK3R2, YWHAG, PXN, HIT1H4C, CDKN2B, CDK1, CDKN2A, CDK6, CREB3, CHD4, HIST1H2BD, HIST1H2BA, TP53*	1.00 × 10^−2^
MAPK signalling pathway	*BRAF, BUSP4, PDGFRA, CACNA1A, HSPA1A, NFKB1, GNA12, MAP4K2, RAC2, FGFR3, CRKL, STK4, MAP2K2, RASA2, MAP3K3, CRK, MAP2K7, FGFR4, PTPRR, TAOK2, PPP3R1, RAF1, MAPK8IP1, MAP2K3, EGFR, PPP3CC, TAB2, TGFB1, MAP3K11, MAPK13, TP53, PPP3CA, AKT2, DUSP10, MAP3K14, JUN, RAPGEF2, MAPK8, MYC, PPM1A, RASGRP4, MAPKAPK3, PRKACA, CACNB1, CACNA1E, MAPT, FGF9, PRKX, DUSP8, CACNA2D4, FGF18, NF1, DUSP7, DUSP3, PRKCB, RPS6KA3, TNFRSF1A, MAP21, STMN1, MEF2C, FGF23, MKNK2, FGFR2, HSPA1B, NFATC3, FGFR1, DUSP16, MAPK1, NFATC1, FGF7, TGFBR2, ELK1, RRAS, DAXX, PDGFRB, ARRB1, PRKACB, TGFB3, CACNB3, NR4A1*	3.94 × 10^−2^
	**miR-138-5p**	
Hepatitis B	*PCNA, CXCL8, CCNA2, SMAD3, BCL2, BIRC5, KRAS, STAT5B, DDX3X, TGF1B, AKT2, CASP3, CCND1, MYC, MMP9, CREB3L2, CCNE1, RELA, CREBBp*	1.05 × 10^−3^
Pathways in cancer	*DVL3, CXCL8, GNAS, ROCK2, SMAD3, BCL2, BIRC5, F2RL3, KRAS, STAT5B, TGFB1, EPAS1, LPAR4, AKT2, PLCG1, CASP3, CCND1, CTNNA1, SKP2, HIF1A, MYC, MMP9, DAPK1, HSP90AB1, GNG2, PTGS2, RARA, CCNE1, GNAI2, RELA, VEGFA, CREBBP, DVL2, PPARD, MDM2, BMP4, PDGFRB, COL4A1*	6.41 × 10^−3^
Notch signalling pathway	*DVL3, APH1A, NOTCH2, HES1, JAG1, DTX1, SNW1, CREBBP, DVL2, MAML1*	3.08 × 10^−2^
p53 signalling pathway	*CCNG1, GADD45A, CASP3, TSC2, CCND1, SHISA5, CCNE1, SERPINE1, GTSE1, MDM2, CCND3*	3.08 × 10^−2^
Viral carcinogenesis	*CCNA2, HIST1H2BK, VAC14, PXN, KRAS, STAT5B, DDX3X, CHD4, HIST1H2BI, CASP3, CCND1, SKP2, SP100, CREB3L2, CCNE1, SNW1, RELA, CREBBP, MDM2, GTF2EI, CCND3, HIST1H2BJ*	4.42 × 10^−2^
	**miR-155-5p**	
Hepatitis B	*FOS, STAT3, NFKB1, CDK4, E2F2, CDK2, SMAD3, BCL2, DDB2, KRAS, CREB1, APAF1, MAVS, MYD88, CCND1, SMAD4, E2F3, PIK3R1, YWHAZ, TBK1, AKT3, PIK3CA, CDKN1A, STAT1, TNF, RELA, IL6, MAPK10*	2.36 × 10^−6^
TGF-beta signalling pathway	*SMAD2, THBS1, PPP2CA, SMAD3, SMAD4, SMAD5, ACVR2A, GDF6, SP1, ACVR1C, PPP2CB, TNF, SMAD1, RPS6KB1*	4.58 × 10^−5^
FoxO signalling pathway	*RS2, STAT3, SMAD2, SETD7, MAPK14, CDK2, PCK2, SMAD3, CAT, EGFR, KRAS, CCND1, SMAD4, S1PR1, GABARAPL1, PIK3R1, SOS1, AKT3, PIK3CA, FOXO3, USP7, CDKN1A, PLK1, SGK3, FOXO1, IL6, IL10, MDM2, MAPK10, C8orf44-SGK3*	1.36 × 10^−4^
Apoptosis	*NFKB1, IL1B, BCL2, PRKAR1B, DFFA, APAF1, MYD88, MAP3K14, TNFRSF10A, PIK3R1, CFLAR, PRKAR2A, TNFRSF10B, PRKAR1A, AKT3, PIK3CA, BIRC3, AIFM1, TNF, RELA, XIAP*	1.51 × 10^−3^
TNF signalling pathway	*FOS, NFKB1, IL1B, MAPK14, VCAM1, TAB2, RPS6KA5, ICAM1, CREB1, CEBPB, MAP3K14, JUNB, PIK3R1, CFLAR, AKT3, PIK3CA, BIRC3, TNF, RELA, IL6, TRAF3, MAPK10*	4.10 × 10^−3^
	**miR-182-5p**	
Fatty acid biosynthesis	*FASN, ACSL4, ACACA*	3.27 × 10^−11^
Viral carcinogenesis	*STAT3, NFKB1, CDK4, NRAS, RASA2, YWHAE, HDAC3, HIST1H2BK, PIK3CB, CREB5, SYK, YWHAG, CCND2, BAX, PKM, CDKN1B, CDK1, CDK6, STAT5B, DDX3X, HIST1H2BD, HDAC9, TP53, EGR3, CREB1, EIF2AK2, SND1, CCND1, SKP2, TBPL2, DDB1, HIST1H4H, PIK3R1, RB1, YWHAZ, RAC1, CDC42, EP300, CREB3L2, CCNE1, PIK3CA, CREB3L1, BAK1, CDKN1A, TBP, RELA, GTF2A1, SRF, CREBBP, GRB2, MAPKAPK2, ATF4, PRKACB, HIST1H4I*	4.00 × 10^−9^
Non-small cell lung cancer	*BRAF, E2F1, CDK4, NRAS, PIK3CB, CDK6, TP53, PLCG1, CCND1, AKT1, PIK3R1, RB1, SOS1, PRKCB, AKT3, PIK3CA, FOXO3, PDPK1, GRB2*	7.78 × 10^−4^
FoxO signalling pathway	*BRAF, STAT3, NRAS, PRKAA2, SIRT1, PIK3CB, SETD7, CCND2, CAT, CDKN1B, IGF1R, GADD45A, CCND1, SMAD4, SKP2, MAPK8, AKT1, IRS4, PLK2, PIK3R1, SOS1, IRS1, PRKAA1, CSNK1E, EP300, AKT3, PIK3CA, HOMER1, FOXO3, CDKN1A, PDPK1, PLK1, SGK3, FOXO1, PLK4, CREBBP, GRB2, BCL2L11*	4.17 × 10^−3^
	**miR-210-3p**	
Other types of O-glycan biosynthesis	*ST3GAL3*	8.99 × 10^−4^
MicroRNAs in cancer	*KIF23, E2F3, RASSF1*	6.28 × 10^−3^
Pancreatic secretion	*CHRM3, RAB27B, ATP2B3*	3.79 × 10^−2^

## Data Availability

The dataset supporting the conclusions of this article is included within the article (and its additional file). Any requests for data and material should be directed to the corresponding author.

## Supplementary Materials

The following supporting information can be downloaded at: https://www.mdpi.com/article/10.3390/ijms26104934/s1.

## Abbreviations

The following abbreviations are used in this manuscript:

TL	Telomere length
rTL	Relative telomere length
TBARS	Thiobarbituric acid
MDA	Malondialdehyde acid
8-OHdG	8′-hydroxy-2′-deoxyguanosine
miRNA	microRNA
WHO	World Health Organization
ROS	Reactive Oxygen species
SD	Standard deviation
CVD	Cardiovascular diseases
