# Supplementary material for: Telomere Length, Oxidative Stress Markers, and Related miRNAs in Non-Invasive Samples of Mild COVID-19 Cases
Source: Int J Mol Sci. 2025 May 21;26(10):4934. doi: 10.3390/ijms26104934 (PMC12111949; doi:10.3390/ijms26104934)
Supplement: Supplementary file 1 [file ijms-26-04934-s001.zip › ijms-3590192-supplementary.pdf]

Supplementary material

Supplementary Table 1. Primers used in the qPCR assay for relative telomere length quantification.

*The reactions involve a final step of dissociation of the amplicons (melting curve) that allows checking the

**Primers**	**Sequence 5´-3´ (Cawthon, 2009)**
Telg	ACACTAAGGTTTGGGTTTGGGTTTG GGTTTGGGTTAGTGT
Telc	TGTTAGGTATCC CTATCCCTATCCCTATCCCTATCCCTAACA
Albu	CGGCGGCGGGCGGCGCGGGCTGGGCGGaa atgctgcacagaatccttg
Albd	GCCCGGCCCGCCGCG CCCGTCCCGCCGgaaaagcatggtcgcctgtt.

**qPCR Reaction Conditions**			
qPCR Tel*		qPCR Albumin*	
95ºC 10min		95ºC 10min	
94ºC 15s<br>49ºC 15s	2 cycles	94ºC 15s<br>49ºC 15 s	2 cycles
94ºC 15s<br>62ºC 10s<br>74ºC 15s	32 cycles	94ºC 15s<br>62ºC 10s<br>74ºC 15s<br>84ºC 10 s<br>88ºC 15s	35 cycles

correct amplification of the analyzed products.

Supplementary Table 2. Targeted enriched Gene Ontology (GO) biological process of the studied miRNAs.

Biological process	GO category	Genes (nº)	p-value
miR-34a-5p			
Protein modification process	GO: 0036211	634	1.20508374063e-41
Biological processes involved in symbiotic interactions	GO: 0044403	182	6.74028581629e-35
Gene expression	GO: 0010467	187	2.01441861777e-34
Viral process	GO: 0016032	162	7.72211567256e-33
Response to stress	GO: 0006950	540	8.60681617999e-15
Cell death	GO: 0008219	232	8.51733082499e-11
Stress-activated MAPK cascade	GO: 0051403	24	6.64819782553e-08
Innate immune response	GO: 0045087	169	3.97462683322e-05
Viral life cycle	GO: 0019058	30	0.00013878095147
miR-138-5p			
Gene expression	GO: 0010467	78	1.54635326126e-26
Biological processes involved in symbiotic interactions	GO: 0044403	49	2.28503722264e-09
Response to stress	GO: 0006950	160	2.76021294526e-09
RNAm metabolic process	GO: 0016071	28	6.06798061733e-09
Viral process	GO: 0016032	43	1.30103358112e-08
Nucleobases-containing compound catabolic process	GO: 0034655	72	9.20186225601e-08
Mitotic cell cycle	GO: 0000278	36	3.70500867943e-07
Notch signaling pathway	GO: 0007219	23	0.000926768952548
Immune system process	GO: 0002376	100	0.00132244412446
miR-155-5p			
Gene expression	GO: 0010467	83	2.20176279433e-20
Response to stress	GO: 0006950	220	3.3065880394e-12
Biological processes involved in symbiotic interactions	GO: 0044403	65	5.35304945047e-12
Fc-epsilon receptor signaling pathway	GO: 0038095	28	8.42313954191e-11
Viral process	GO: 0016032	56	2.43948118829e-10
Immune system process	GO: 0002376	143	1.26028225569e-05
miR-182-5p			
Gene expression	GO: 0010467	158	6.60653952898e-46
Biological processes involved in symbiotic interactions	GO:0044403	142	2.80250302877e-38
Viral process	GO: 0016032	127	7.10769952814e-36
Response to stress	GO: 0006950	371	1.96552866717e-18
Cell death	GO: 0008219	179	1.10588669707e-17
mARN metabolic process	GO: 0016071	58	4.5050272711e-17
miR-210-3p			
Organelle (cellular component)	GO: 0043226	40	0.00521785710312
Ion binding (Molecular function)	GO: 0043167	27	0.0429338374573

Supplementary Figures

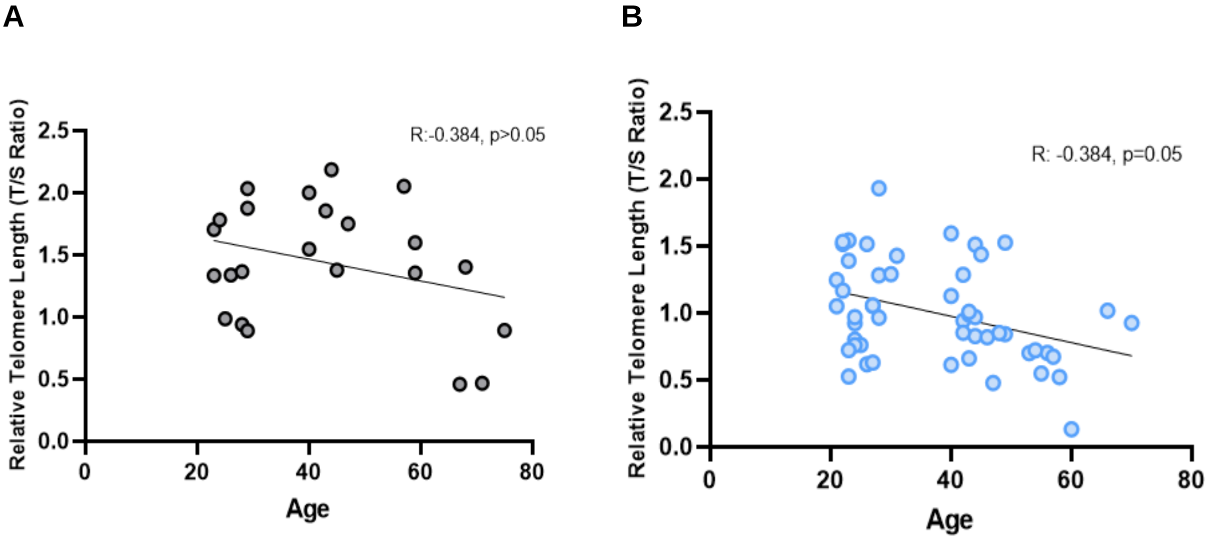

Supplementary Figure 1. Correlation of rTL and age in the non-COVID-19 control group (**A**) and COVID-19 cases **(B**).
